# ALF-Score++, a novel approach to transfer knowledge and predict network-based walkability scores across cities

**DOI:** 10.1038/s41598-022-17713-y

**Published:** 2022-08-18

**Authors:** Ali M. S. Alfosool, Yuanzhu Chen, Daniel Fuller

**Affiliations:** 1 Department of Computer Science, Memorial University of Newfoundland, St. John’s, Canada; 2 School of Computing, Queen’s University, Kingston, Canada; 3 Department of Community Health and Epidemiology, College of Medicine, University of Saskatchewan, Saskatoon, Canada

**Keywords:** Computer science, Mathematics and computing, Scientific data

## Abstract

Walkability is an important measure with strong ties to our health. However, there are existing gaps in the literature. Our previous work proposed new approaches to address existing limitations. This paper explores new ways of applying transferability using transfer-learning. Road networks, POIs, and road-related characteristics grow/change over time. Moreover, calculating walkability for all locations in all cities is very time-consuming. Transferability enables reuse of already-learned knowledge for continued learning, reduce training time, resource consumption, training labels and improve prediction accuracy. We propose ALF-Score++, that reuses trained models to generate transferable models capable of predicting walkability score for cities not seen in the process. We trained transfer-learned models for St. John’s NL and Montréal QC and used them to predict walkability scores for Kingston ON and Vancouver BC. MAE error of 13.87 units (ranging 0–100) was achieved for transfer-learning using MLP and 4.56 units for direct-training (random forest) on personalized clusters.

## Introduction

Walkability is a concept that many researchers have used to operationalize characteristics of the environment that support walking. Although there are multiple conceptual definitions of walkability in the literature^1–7^ there is no single agreed-upon operational definition of walkability. There are a number of existing walkability measures that provide walkability scores for Canada each with different strengths and limitations. While a number of city-specific walkability measures have been developed, there are two prominent, national-level walkability measures available in Canada: Walk Score and the Canadian Active Living Environments measure (Can-ALE). These measures each has different strengths and limitations. Both Walk Score and Can-ALE are heavily used/cited^8–11^. But there are some noticeable drawbacks and opportunities to improve these measures. These limitations are important and are the result of limited interdisciplinary work between the fields of computer science, public health, and urban planning. The important limitations of previous works^12–16^ include: incomplete use of road structures, lack of predictive models, low spatial resolution, lack of user opinion, lack of personalization, and limited transferability to new cities. For the purpose of comparison in this paper, we choose Can-ALE^17^ as it is commonly used by researchers and end-users alike.

In our previous paper, we created the Active Living Feature Score, ALF-Score^18^, a completely new approach to measure walkability. This predictive approach allows us to use various important features currently not utilized by most existing walkability measures, such as road network structure as nodes, road embedding, complex networks centrality measures, and user opinion along with a new approach of using machine learning to estimate walkability scores. By using a predictive approach ALF-Score is able to generate walkability scores with high spatial resolution allowing us to predict walkability scores for any point along a road network. To briefly explain these features, road embedding generally refers to a way to represent road networks as vectors and can also help reduce dimensions of the network while capturing the topology of the network. Centrality measures on the other hand aim to find important nodes/edges in networks under various criteria, assumptions and using different methods and techniques.

Moreover, in our paper ALF-Score+^19^ which followed after ALF-Score, we showed an extension of ALF-Score which utilizes user and system defined user-demographics to create individual sociodemographic profiles to develop profile clusters. User labels and profile clusters are then used by ALF-Score’s pipeline to generate machine learning predictive models capable of estimating personalized walkability scores specific to each profile cluster. Examples of cluster profiles include for example groups of volunteer participants with similar or varying demographics who may have a similar view of walkability. For example, female professionals in their 20’s and 30’s who do not live alone and have no children who perceive walkable distances as being greater than 1500 meters, may perceive walkability similarly and can form a profile cluster.

In this paper, we introduce ALF-Score++ which is another extension of ALF-Score. ALF-Score++ focuses on transferability. The overall goal of ALF-Score++ is to ensure the pipeline is capable of generating reproducible predictive walkability models that are transferable and able to generate walkability scores for new cities without the need for any new user data (zero-user-input approach, further explained in methods section) or training.

Our main objective is to ensure our pipeline can generate transferable models. Transfer learning is yet another missing technique from many of the existing walkability measures. Being able to generate reproducible and transferable predictive walkability models is an important component of which ALF-Score++ takes advantage of in two ways: (1) by gaining the ability to utilize previously learned knowledge when directly generating walkability scores for new cities (zero-user-input), (2) by using this previously learned knowledge as a base to train new models which can lead to reduced training time, improved accuracy, reduced resource consumption, and reduction in the labels required for supervised learning tasks. A well generalized model will have the capability of transferring its knowledge to various cities never seen during its training to generate accurate walkability scores in a fraction of the time without the need for any new user input within the target city.

In this paper we will highlight application of ALF-Score++ to three new cities of Kingston Ontario (ON), Vancouver British Columbia (BC), and Montréal Quebec (QC).

## Background

**Figure 1:**
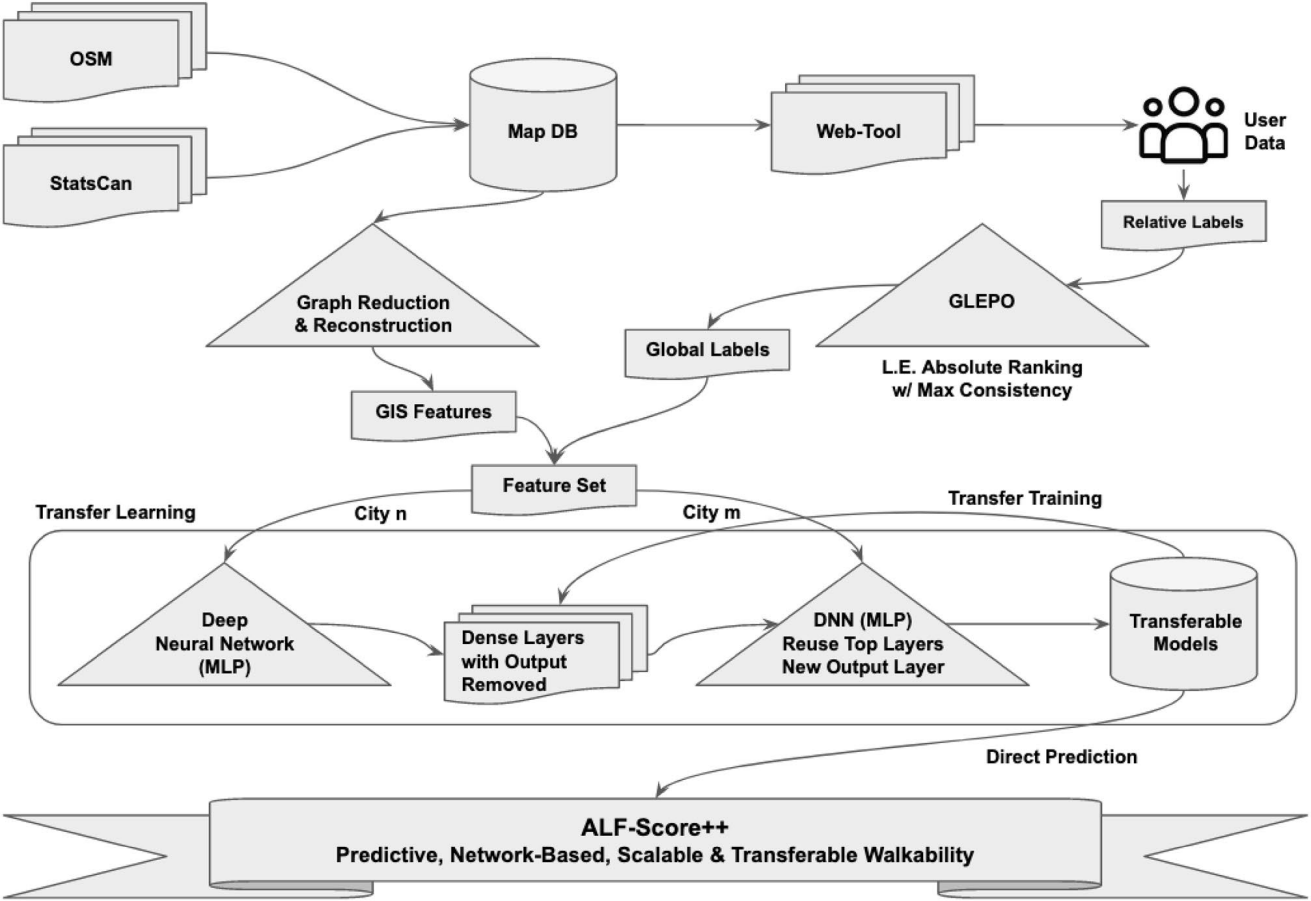
ALF-Score++ utilizes features similar to that of ALF-Score and ALF-Score+ such as road network structure, POI, centrality measures and road embedding. GLEPO’s linear extension of user opinions^18^ that produces a global view of relative user opinions, is then aligned with the features as an input to the machine learning processes. Models trained by ALF-Score++ are applicable to cities previously seen and unseen by the algorithms during the training processes. Walkability estimates that are produced through trained models will have a high spatial resolution, be representative of user opinion and provide a better insight of different regions and neighbourhoods. (Figure drawn by the authors).

Ensuring ALF-Score’s pipeline (Fig. 1) does not engage in repeated wasteful activities is one of the sub-objectives of this research. This is particularly important since road networks can vary in size with some cities being very small (e.g. with a population of a few hundred) while some other cities could be very large and dense (eg. Tokyo, Japan with a population of over 37 million people in just one city). Table 1 shows a list of various cities alongside their road network size, number of points of interests (POIs), population and total land area size. Processing data from St. John’s, NL as opposed to data from Toronto, ON will have significantly different resource requirement and time consumption due to the change in the size of the city leading to an extended set of complexities introduced into the network. If the algorithms are not optimized, this difference in requirements may lead to infeasibility of the research. In this research we have experimented with all cities mentioned in Table 1; however, we will only highlight the results for Kingston ON, Vancouver BC, and Montréal QC.

**Table 1:** List of road networks for various cities with their network and POI sizes that have been experimented with in this research.

City	# of nodes	# of edges	# of POIs	Population density	Total land area (km^2^)
Victoria, BC	6770	8593	3318	85,792	19.47
Kingston Metro, ON	3427	4769	813	161,175	1906.82
St. John’s Metro, NL	5364	6851	592	205,955	804.63
Vancouver Metro, BC	45,125	60,299	13,321	2,463,431	2878.52
Montréal Metro, QC	76,663	114,414	10,045	4,247,000	4604.26
Toronto Metro, ON	479,520	Over a million	23,930	6,417,516	5905.71

For brevity, in this paper we mostly focus on 3 cities of Kingston, Vancouver and Montréal. Nodes and edges are extracted from road networks. Population density and the total land area information are excerpted from Wikipedia.

Transfer learning is the process of re-utilizing the knowledge learned from a task in other tasks. In many machine learning approaches, solving a single task at hand has been the main focus, but now development of approaches that help with transfer learning has become a very popular focus in the recent years^20^. As with most real-world problems, specifically in machine learning, collecting labelled data is a time consuming, expensive^21^ and difficult task. Transfer learning uses the knowledge learned from previous problems to solve new but related problems^22^. As a result of its approach, transfer learning can help reduce training time, resources and the required labeled data^23^ as well as improve overall accuracy. Weiss et al.^24^ provide a much more formal definition of transfer learning as the following: “given a source domain ${\mathscr {D}}_{\text {S}}$ with a corresponding source task ${\mathscr {T}}_{{\mathscr {S}}}$ and a target domain ${\mathscr {D}}_{\text {T}}$ with a corresponding task ${\mathscr {T}}_{{\mathscr {T}}}$, transfer learning is the process of improving the target predictive function $f_T(\cdot )$ by using the related information from ${\mathscr {D}}_{\text {S}}$ and ${\mathscr {T}}_{{\mathscr {S}}}$, where ${\mathscr {D}}_{\text {S}} \, \ne {\mathscr {D}}_{\text {T}}$ or ${\mathscr {T}}_{{\mathscr {S}}} \, \ne \,{\mathscr {T}}_{{\mathscr {T}}}$”.

The general idea behind transfer learning is to apply a model that was previously trained on labelled data (in case of supervised learning) to another similar task with little data available and instead of starting from scratch, start with some existing knowledge and captured patterns. Transfer learning is typically used in computer vision. For example, the weights of a model that was trained to detect apples could be transferred to another task of detecting fruits. In this case, instead of training the new model to detect apples from scratch, the knowledge about detecting apples are transferred and the algorithm now looks to learn how to detect other fruits. Transfer learning is a technique that not only requires significantly less data for training, but it will also speed up the training process^25^.

Transfer learning falls under representation learning with the goal of using the same representation in various tasks. According to Ian Goodfellow^26^ transfer learning can be viewed as a particular form of multi-task learning where it normally revolves around supervised learning. Although, transfer learning can also be used to solve unsupervised learning tasks. The goal of transfer learning is to take advantage of previously trained models and to extract knowledge that would be useful in the new task. However, transfer learning is also very useful to directly generate predictions in another environment and for other tasks without any more learning^26^. There are a few approaches to transfer learning including feature extraction, training generalized models, and use of existing pre-train models. When it comes to feature extraction, determining the best representation for the problem at hand is a key task which if done properly, can often lead to much better and more accurate results. Carefully selected features can often lead to a powerful and well-generalized model that can be applied to various related problems. Furthermore, using already available pre-trained models is yet another, very popular option. In fact there are numerous pre-trained models available online that provide ready weights for many popular tasks such as classifying certain types of images, object detection and object tracking. It is important to highlight that this approach only requires access to a previously trained model, and not the entire dataset. Additionally, another approach to solve a task using transfer learning where there is not enough data available and no pre-trained models can be found, is to take the previous approach a step further and train models that are designed for another but similar task and that have an abundance of data. These models can then act as a starting point to address the original task. To highlight the difference with the previous approach, in this technique to solve task A, we will be doing our own training on a similar task B. Once we are satisfied with the model, we can now transfer and reuse this knowledge. Goodfellow in his book^26^ further discussed two extreme forms of transfer learning, namely: (1) one-shot learning — which only one labeled example of the transfer task is given while, (2) zero-shot learning, has no labeled example given.

## Methods

ALF-Score++ is the second extension of ALF-Score pipeline with a focus on transferability. ALF-Score++ pipeline (Fig. 1) utilizes a map database that contains road network data as well as POIs extracted from Statistics Canada^27^ and OpenStreetMap (OSM)^28^ respectively. The map database feeds into two separate processes: (1) GIS feature extraction, (2) user data extraction through a web-tool interface. The GIS feature extraction process extracts and generates the required features such as node lists, edge lists, various centrality measures, road embedding, and various POI features. The output of this process is fed into the machine learning component as one of its three main input feature sets. User data extraction process involves a web-tool interface that utilizes road data to feature various points on an interactive map where users provide their opinion and data. User data is broken into two separate processes, each of which will result in a separate input to the machine learning component. The first process is the collection of user opinions through the web-tool where users provide relative ranks for various points on the map. This process passes the user opinion to our Generalized Linear Extension of Partial Orders or GLEPO^18^ algorithm to convert users’ relative ranks to a globalized rank among all submissions. The output of GLEPO is fed to the machine learning component as it’s second feature set. This input serves as the *y* label vector during the training and testing processes. The second process of user data revolves around their demographics. This process uses various clustering techniques and unsupervised learning methods to generate profile clusters. These profile clusters represent users deemed by the algorithm as similar. These profile clusters are then fed into the machine learning component as its third feature set. The machine learning component utilizes these three feature sets in conjunction with its internal transfer learning process and the general flow is as follows. GIS features form a feature set and are then associated with specific locations that have their rankings available through the GLEPO algorithm. This feature set is in the form of {features, label}: {x, y}, where *x* represent an entry of features  and *y* represent the label. The expectation from the trained models is that they will produce a prediction given an unlabelled set such as {features, ?}: {x, ?} where ? would be replaced with $y'$ prediction. These models will be trained on the data from only one specific city. The first round of models trained through a deep neural network technique will then be used to transfer their knowledge to the second round of training where transfer learning utilizes appropriate layers while replacing the output layer. The new data used in the transfer learning process will then include features and user opinion from a second city. The output will be a more generalized model capable of transferring its knowledge to cities never seen during its training process. The personalization process utilizes this transfer learning approach to do the same task but on each separate profile cluster, resulting in multiple models capable of predicting personalized walkability scores for cities seen or never seen by the algorithm.

### Data preparation

As an overview to the data used in this research, the general structure of our road network and feature set remains the same to one described in our paper ALF-Score^18^. We collected a small set of user opinion data containing 1050 user entries covering 895 unique locations for the city of St. John’s, NL. This includes $n = 40$ users with $n = 20$ (50 %) women with an average age of 48.6 (standard deviation = 17.1). The most commonly reported walkable distance was 800–1000 meters while ten participants (25%) reported living alone whereas 14 participants (35%) reported living with children with the average number of children being 2.6 (standard deviation = 1.2). The most commonly reported professions were Retired $n = 8$ (20%), Professor $n = 4$ (10%), and Nurse $n = 4$ (10%). In addition, more user opinions have been collected which are specific to the city of Montréal, QC containing 785 user entries covering 775 unique locations. Similarly, this includes $n = 21$ users with $n = 13$ (62%) men with an average age of 40.95 (standard deviation = 17.29). The most commonly reported walkable distance was 1200–1400 meters while five participants (24%) reported living alone whereas 8 participants (38%) reported living with children. The most commonly reported professions were Professional $n = 4$ (19%), Professor $n = 3$ (14%), and Retired $n = 3$ (14%).

Participation in the crowd-sourced data collection is completely voluntarily. All participants provide their informed consent online through our online crowd-sourcing web-tool. The consent form is the first thing participants see on the web-tool. Participants are informed that their participation is voluntarily. A popup appears when accessing the web-tool informing volunteer participants that by submitting the form on our online crowd-sourcing web-tool, they confirm they have fully read and understood our consent form and privacy notes and have consented to participate in this study and have their data collected and used in the research. Relevant information such as the informed consent form as well as our privacy notes are visibly and clearly available via the popup window and throughout the web-tool. Participants are notified online through the popup and footer content that user submission will automatically be considered as their consent to participate. The proposal for this research has been reviewed by the Interdisciplinary Committee on Ethics in Human Research (ICEHR) and found to be in compliance (ICEHR Approval Number: 20220406-SC) with Memorial University’s ethics policy and in accordance with the Tri-Council Policy Statement on Ethical Conduct for Research Involving Humans (TCPS2), the project has been granted full ethics clearance. This study did not include any minors.

To prepare the map database, the first step is to gather the feature set that includes various information such as POI, road embedding and road network data. The POI data is available freely through OpenStreetMap (OSM)^28^. We utilized Overpass-Turbo^29^, with the help of a customized extraction code, to extract OSM POIs from 53 unique amenity categories. Once complete, we devised a new algorithm that creates POI-based features for all nodes within the network. Below is an example of a single POI contained within a GeoJSON file extracted from OSM through Overpass-Turbo. Each POI point is divided into 2 parts: (1) description, (2) geometry. Description contains the type and properties of the point while the geometry contains location’s type as well as its coordinates:

- “type”: “Feature”, “properties”: “id”: “node/1401297904”,
  “amenity”: “fire_station”, “name”: “Caserne 29 Rosemont”
  “geometry”: “type”: “Point”, “coordinates”: [ -73.5762681, 45.5453509 ]

As each POI is represented by a node on the road, we assign a value to 10 separate distance ranges which represent the number of POIs of a specific category within a specific distance range to a specific node. Based on 53 amenity categories, we can produce a POI feature list containing 530 feature columns and *n* rows for the number of unique nodes in the road network. Below is an example of one possible POI feature header structure followed by an example of a single entry for a unique node:

- node_id lon lat bar_200 bar_400 bar_600 ... bbq_200 bbq_400 bbq_600 ...
  317 -73.57113438 45.51020696 0 6 12 ... 14 11 12 ...

While road network data is available freely from both OSM as well as Statistics Canada^27^, we chose to extract them from Statistics Canada in the form of ArcGIS Shapefile^30^. The Shapefile for the entire Canada was extracted for the year 2016 which is the most recent Census year available at the time of this research. Furthermore, QGIS^31^ which is “a free and open-source cross-platform desktop geographic information system application that supports viewing, editing, and analysis of geospatial data”, was used to extract road networks specific to four different cities of St. John’s NL, Montréal QC, Vancouver BC and Kingston ON from the single large Shapefile containing the road network for the entire Canada. All of the individual city sub-networks were further processed to build specific node lists and edge lists which are used in various locations within the pipeline. We utilize “shp2graph” package^32^ through R^33^ to generate node lists and edge lists for our road networks which have been stored in the form of graphs. It is important to mention that depending on the data source and the format, the geographical coordinate systems may differ . For instance, some formats may present coordinates in UTM^34^ or WGS^35^ while others may present them in different coordinate systems. Appropriate conversions, where applicable, may be required. As is the case with many researches, it is absolutely crucial to maintain a unified unit of measurement throughout the research to avoid any unwanted disasters^36^. Individual city sub-networks are also processed to generate various complex networks features such as different centralities as well as road embedding features for all road networks. Furthermore, when working with large networks, graph reduction and reconstruction techniques^37^ may be applicable. Additionally, the edge list for each city is processed through Cytoscape^38^ which is “an open source bioinformatics software platform for visualizing molecular interaction networks and integrating with gene expression profiles and other state data”, to generate a list of network features. To generate road embedding features, edge lists are processed through Node2vec^39^ which is “an algorithm to generate vector representations of nodes on a graph”. All features that are not numerical go through an encoding processed called one-hot encoding to prepare the features for our machine learning processes.

The next step needed to take to prepare the pipeline is the application of Generalized Linear Extension of Partial Orders or GLEPO^18^. GLEPO requires a few data sets such as user opinion, node list and a distance matrix connecting all the nodes within the network. The overall GLEPO pipeline involves multiple algorithms such as *seperateBySub* which is used to prepare user opinions into subsets that are suitable for processing. Various other algorithms such as *calculateDistance*, *FindDistance*, *addToSorted*, *FindVLink*, *RandomizeInsertion*, *normalize* and *GLEPO* are also used to further process user opinion and to convert their relative rankings into generalized scores which are globalized among all opinions. The output of the GLEPO pipeline is a generalized list of user opinion which can be fed into the next pipeline. This globalized list is crucial to the entire structure of ALF-Score as it plays the important role of ground truth used in the machine learning component.

### Experiments

There are three main experimentation scenarios used to guide this research forward, and they are: (1) matching approach, (2) combined approach, (3) zero-user-input approach.

**Matching approach** is a scenario where users' opinions from a specific city are used to train and test models for the matching city. This approach is an important base to our machine learning pipeline and focuses on testing the feasibility and accuracy of the pipeline derived from users' opinions and feature set belonging to the same city. For instance, using users' opinions and feature set collected for the city of St. John’s, NL to train and test models on St. John’s. Furthermore, this approach is used for testing the scalability of the model to ensure process stability when it comes to very large road features and user opinion data.

**Combined approach** is an approach that focuses on transferability of ALF-Score++’s pipeline. This approach uses data from multiple cities to train and test models. These models can then be applied to cities either seen by the pipeline during the training process or cities never seen by the algorithms before. This approach aims to test and verify that transfer learning can improve the overall generalization of the models while broadening models’ applicability. There are multiple variations in this scenario, specifically how the training and the testing sets are selected. Two of our commonly used variations are random and semi-random selections. In the random selection, a typical 80–20% train-test distribution is used that includes data from two cities. In the semi-random approach, 50% of the data for only one city is randomly selected for testing purposes whereas the remaining 50% is combined with the entire data from the second city to form the training set. The model is tested on both cities.

**Zero-user-input approach** aims to use models that are previously trained on specific city/cities to predict walkability scores of other cities. This approach takes advantage of predefined features and pre-trained models to generate walkability scores for points in cities never seen by the algorithms. This approach is very important to help us identify how applicable and transferable are the pre-trained models to data from unseen cities and whether the patterns observed and learned in different cities are similar and transferable to one another. Models in this scenario could have been trained on either a single city or be multi-city models. The models in this scenario can be applied to data form either cities never used in the training process or previously trained cities, making them very versatile.

### Transfer learning

ALF-Score^18^ pipeline has tested for various supervised and semi-supervised approaches and methods. However, the most promising shallow models are random forest, support-vector machine (SVM) and decision tree whereas the most promising deep model was multi-layer perceptron neural network (MLP). All of these methods generated reasonable accuracy results while random forest performing the best among all. We set up random forest with 100 estimators (the number of trees in the forest) while its maximum depth of the tree was not limited. Most other parameters such as the number of jobs to run in parallel, the number of features to consider when looking for the best split and bootstrap sampling were set to scikit-learn^40^ default parameters. Random forest is an ensemble approach. Ensemble learners aim to use multiple weak learners to build a strong learner that perform very well taking a divide and conquer approach. Random forest uses standard decision tree which could be considered as its weak learner. Multiple of these trees will then form a forest which can perform better as a group. Random forest performs significantly better. There are two specific functions in scikit-learn’s random forest that although not specifically labeled as transfer learning approaches, are geared toward transferring previously learned knowledge. These functions are *warm_start* and *partial_fit*. Warm start aims to fit an estimator repeatedly over the same data set but with varying parameters. Using this approach one can look at various parameters to improve performance while reusing the model learned from previous parameters to save computing resources and time. Warm start is typically used for fine tuning the model parameters. Partial fit on the other hand aims to provide an online machine learning approach while maintaining a fixed model parameters between calls, by allowing for new data in every call. This data is called mini-batch. Online machine learning is a method used to update the predictor in a sequential order as new data becomes available. This is the opposite approach taken in batch learning where the training data set never changes.

Furthermore, MLP was used as a way to utilize deep learning specifically as a doorway to transfer learning. In this paper, we work with transfer learning under the assumption that previously trained models of similar task are available (through ALF-Score). The first step to initiate the transfer learning process is to import three sets of data: (1) previously trained MLP models, (2) GIS features such as POI, centrality and embedding features associated with the new city, (3) user data such as user opinion and demographics associated with the new city. After a successful import of the data, the usual data processing and preparation steps will need to be taken, such as dealing with incomplete entries and processing features through one-hot encoding, where applicable. In this research we use TensorFlow^41^ to facilitate MLP training and transfer learning processes. TensorFlow is “a free and open-source software library for machine learning and artificial intelligence” that enables us to apply various techniques with very efficient implementations. To set up TensorFlow for transfer learning, the first step is to create a Sequential model. Next we can add multiple Dense layers as our hidden layers. Each dense layer takes in a unit value and an activation function. The unit value which is a positive integer defines the dimensionality of the output space. The activation function^42^ acts as a trigger based on the input values and fires only if input exceeds a set threshold. In this setup, we use ReLU activation function^43^. If the input is negative, ReLU returns 0, otherwise it will return the actual input. For the last layer that acts as our output layer, the unit is set to 1. It is common to see Softmax activation function being used in classification tasks for the last dense layer, however, since our task is a regression problem we use linear activation function. At this point, the model needs to be compiled with the loss function, optimizer and metrics set. We set the loss function to *mean_absolute_error*, the optimizer to *adam* and the metrics to *mean_squared_error*. The last step is to fit the model by passing the feature set followed by the labels and setting the number of epochs and the size of the validation split. Depending on the batch size, number of epochs and the size of data, the process may take a while. This process will result in a model trained on the {features, label}: {x, y} set. In our approach, we only import the models previously trained through this approach.

ALF-Score uses various combinations of dense layers and number of neurons. Table 2 shows a brief set of example settings we have experimented with. To transfer the model generated/imported as above, the first step is to create a new Sequential model and copy the hidden layers desired from the original model over to the new model. In the process we will exclude the output layer. We also need to ensure all transferred layers are frozen by setting them as non-trainable so the algorithm will not modify them. Next, we add a dense output layer to the new model with unit set to 1 and activation function set to linear. Finally we set the loss function to *mean_absolute_error*, the optimizer to *adam* and the metrics to *mean_squared_error* and compile and fit the new model. After a few iterations/epochs, we can try to unfreeze the reused hidden layers to allow back propagation to modify and fine-tune them and re-evaluated the performance. It is also suggested^26^ to reduce the learning rate to avoid changes in weights that are fine-tuned when these layers are unfrozen. A good rule of thumb is to train the model for the new task for a few epochs while the reused layers are frozen. Then unfreeze the reused layers and continue to train, with reduced learning rate, for further fine-tuning these layers. When talking about transfer learning, learning rate is always an important variable to consider. If the learning rate is set too high, training may diverge and if the learning rate is set too low, the processing speed will be very slow to reach a convergence. Experimenting with various parameters may be a good approach to find the best setting that may be most appropriate in a particular task.

**Table 2:** Various deep neural network settings under which MLP and transfer learning were experimented with.

# of dense layers	Output shape range	Total parameters	Optimizer	# of epochs
2	8–16	10,945	Adam	200
5	50–300	418,301	Adam	300
11	50–1000	2,673,301	AdaMax	400
12	50–800	2,303,001	AdaMax	600

In this research, three methods are used to measure performance, specifically, mean absolute error (MAE), root mean squared error (RMSE) and coefficient of determination ($R^2$). Mean absolute error (MAE) is defined as $MAE = \frac{\sum ^n_{i=1} |y_i - x_i|}{n}$ where $x_i$ is the actual value, $y_i$ is the prediction, and *n* is the total number of data points. Root mean squared error (RMSE), on the other hand, is defined as $RMSE = \sqrt{\frac{\sum ^n_{i=1} (x_i - y_i)^2}{n}}$. Furthermore, $R^2$ is defined as $R^2 = 1 - \frac{\sum (y_i - x_i)^2}{\sum (y_i - \hat{y})^2}$ where $\hat{y}$ is the mean value of *y* and $R^2$ can range in $(-\infty ,1]$ with values close to 1 showing better performance.

It is important to note that if the input data of the new task does not have the same shape structure as the data used in the original task, they will need to be processed to match the original size. However, this is not the case with ALF-Score++ since the structure of feature sets used for training various models remain the same. Additionally, according to Géron^25^ “...transfer learning will work best when the inputs have similar low-level features”. To further expand on this, high-level features are typically more useful in a new task and are generally found in the upper hidden layers. They may be useful to their original task but likely there may not be much relevancy between upper layers from the original task with the new task. So it is a good idea to replace these layers for the new task as they will likely be very different to that of the original task. This however, is not always the case and varies from task to task. For example, a voice recognition task will still need to produce the correct and valid words associated to its output layer. However, top layers may need to recognize words spoken by different people. In this case, reusing the top layers may be more useful^26^. Furthermore, typically the output layer of the original model will be replaced since it is no longer useful as we seek to update the output using the new input. It should be noted here that it is suggested that the more similar the tasks are, the more hidden layers may be reused. For instance, in case of ALF-Score++ since the original task is very similar to the new one, we can try by keeping all hidden layers and only replace the output layer.

## Results

**Figure 2:**
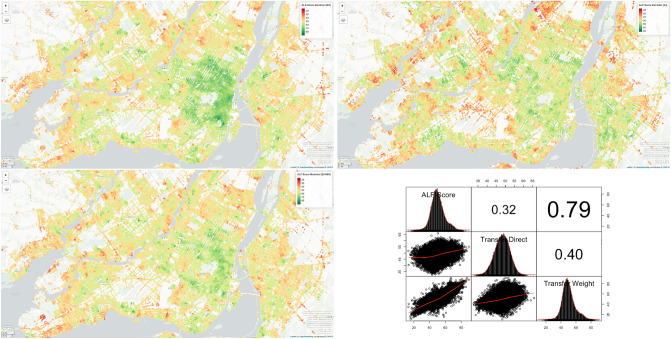
Walkability results produced by 3 separate variations of ALF-Score and ALF-Score++ for the city of Montréal, QC and their correlation. Top left: predictions based on a model only trained for Montréal’s user data. Top right: predictions based on a transferred model only trained for a single city’s user data (St. John’s). Bottom left: predictions based on a model trained for Montréal’s user data while having the previously trained weights for St. John’s user data transferred in its transfer learned training process. Bottom right: correlation between the three variations. The road network for Montréal maintains over 76 thousand nodes. ALF-Score’s walkability scores range between 0 and 100 units. This range can be adjusted if needed. (Maps generated through RStudio^44^ Version 1.2 using mapview package from rstudio.com. Correlation figure generated through RStudio^44^ Version 1.2 using PerformanceAnalytics package from rstudio.com).

In this research, we were able to successfully achieve transferability for ALF-Score++. First, using the newly collected user opinion data for the city of Montréal QC, we were able to achieve a consistency of 99.6% during the GLEPO processing stage. While various feature combinations and machine learning techniques were experimented with, we were able to achieve our lowest prediction MAE error (Matching approach) using random forest shallow model at 11.87 units (Fig. 2 top left) while MLP was the best performing deep model with an MAE error of 13.87 units.

**Figure 3:**
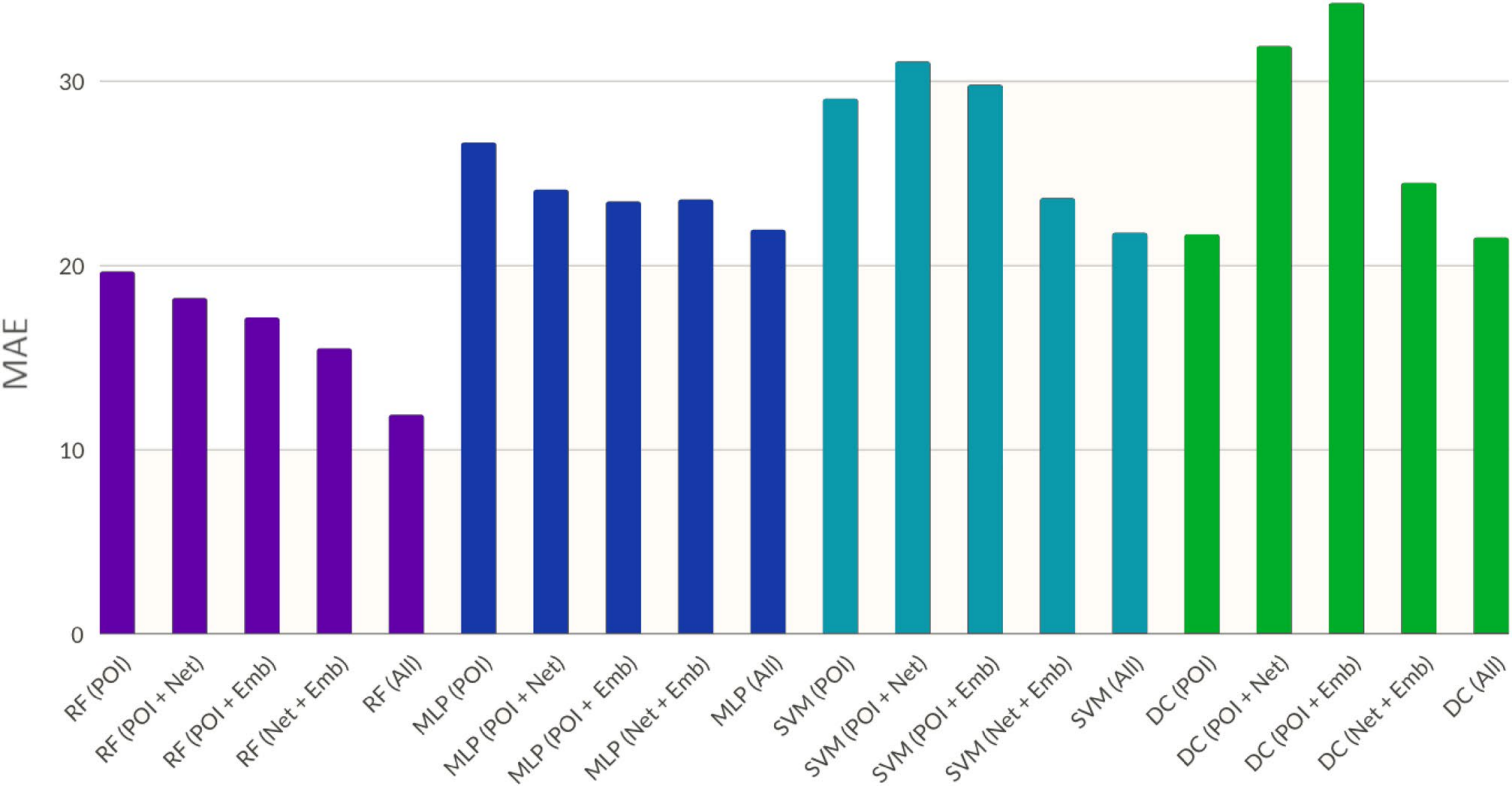
Experimentation results of four machine learning techniques over five feature combinations for the city of Montréal, QC with a data split of 80–20. The bars represent MAE error over a range of 0–100 units. *RF:* random forest, *MLP*: multi Layer perceptrons, *SVM*: support vector machine, *DC:* decision tree. RF provides the best performance overall. (Bar plot generated through matplotlib^45^ Version 3.4.3 from matplotlib.org).

Figure 3 and Table 3 highlight some of the techniques and feature combinations used to generate ALF-Score for the city of Montréal using the user opinion data collected from the same city. Random forest, using all features produces the best results with the least MAE.

**Table 3:** Exploration of various machine learning techniques and feature combinations over an 80–20 data split (matching approach) for the city of Montréal, QC reflecting their top performing accuracy.

Technique	POI	POI +	POI +	Network +	All
		Network	Embedding	Embedding	
**Mean absolute error (MAE)**					
Random forest	19.65	18.20	17.13	15.47	**11.87**
MLP	26.65	24.08	23.44	23.56	21.91
SVM	29.03	31.04	29.78	23.63	21.74
Decision tree	21.65	31.87	34.23	24.45	21.49
**Root mean squared error (RMSE)**					
Random forest	24.75	22.19	22.61	20.28	**14.74**
MLP	29.99	28.09	26.81	25.18	22.69
SVM	34.91	35.66	35.88	27.17	25.09
Decision tree	36.73	34.57	36.67	27.02	25.53
**Coefficient of determination (**$R^2$**)**					
Random forest	0.7291	0.7590	0.7516	0.7784	**0.8314**
MLP	0.6386	0.6892	0.7076	0.7203	0.7520
SVM	0.6388	0.6258	0.6257	0.7051	0.7172
Decision tree	0.6014	0.6210	0.6079	0.7020	0.7249

Results represent MAE and RMSE errors over a range of 0–100 units, as well as $R^2$.

Significant values are in [bold].

**Table 4:** Exploration of the three experimentation approaches (1) matching, (2) combined and (3) zero-user-input over 5 different feature combinations and 2 different data split approaches based on data from the cities of St. John’s NL and Montréal QC reflecting MAE error range of 0–100 units.

MLP	POI	POI +	POI +	Network +	All
		Network	Embedding	Embedding	
St. John’s (STJ on STJ (100%))$^{(1)}$	27.55	26.22	22.23	21.91	17.88
Montréal (MTL on MTL (100%)$^{(1)}$	26.65	24.08	23.44	23.56	21.91
STJ on MTL (100%)$^{(3)}$	n/a	n/a	n/a	n/a	32.44
STJ on STJ (50%) + MTL (100%)$^{(2)}$	26.87	25.10	23.55	19.31	15.77
STJ on STJ + MTL$^{(2)}$ (rand 80-20)	25.87	23.74	21.45	20.23	**14.12**
MTL on STJ (100%)$^{(3)}$	n/a	n/a	n/a	n/a	33.89
MTL on STJ + MTL$^{(2)}$ (rand 80-20)	25.11	22.23	21.67	20.11	16.23
MTL on STJ (100%) + MTL (50%)$^{(2)}$	27.67	24.86	14.43	21.51	16.73
MTL on STJ (100%) + MTL (80%)$^{(2)}$	24.84	20.17	19.92	18.36	**13.87**
MTL on STJ (100%) + MTL (20%)$^{(2)}$	29.66	25.34	25.73	22.89	18.34

Significant values are in [bold].

**Table 5:** Exploration of the three experimentation approaches (1) matching, (2) combined and (3) zero-user-input over 5 different feature combinations and 2 different data split approaches based on data from the cities of St. John’s NL and Montréal QC.

MLP	POI	POI +	POI +	Network +	***All***
		Network	Embedding	Embedding	
**Root mean squared error (RMSE)**					
St. John’s (STJ on STJ (100%))$^{(1)}$	30.14	29.06	26.82	25.37	20.13
Montréal (MTL on MTL (100%)$^{(1)}$	29.99	28.09	26.81	25.18	22.69
STJ on MTL (100%)$^{(3)}$	n/a	n/a	n/a	n/a	38.79
STJ on STJ (50%) + MTL (100%)$^{(2)}$	30	28.64	25.05	23.13	18.67
STJ on STJ + MTL$^{(2)}$ (rand 80-20)	28.21	26.92	25.63	24.22	**17.91**
MTL on STJ (100%)$^{(3)}$	n/a	n/a	n/a	n/a	40.33
MTL on STJ + MTL$^{(2)}$ (rand 80-20)	28.84	25.84	25.36	23.87	19.17
MTL on STJ (100%) + MTL (50%)$^{(2)}$	31.02	27.9	27.11	24.69	19.71
MTL on STJ (100%) + MTL (80%)$^{(2)}$	27.31	24.77	24.82	22.23	**17.98**
MTL on STJ (100%) + MTL (20%)$^{(2)}$	34.17	30.06	29.75	26.28	22.93
**Coefficient of determination (R**^**2**^**)**					
St. John’s (STJ on STJ (100%))$^{(1)}$	0.6674	0.6773	0.7039	0.7219	0.7676
Montréal (MTL on MTL (100%)$^{(1)}$	0.6779	0.6993	0.7080	0.7180	0.7475
STJ on MTL (100%)$^{(3)}$	n/a	n/a	n/a	n/a	0.5840
STJ on STJ (50%) + MTL (100%)$^{(2)}$	0.6771	0.6959	0.7174	0.7455	0.7755
STJ on STJ + MTL$^{(2)}$ (rand 80-20)	0.6846	0.7185	0.7150	0.7344	**0.7958**
MTL on STJ (100%)$^{(3)}$	n/a	n/a	n/a	n/a	0.5756
MTL on STJ + MTL$^{(2)}$ (rand 80-20)	0.6795	0.7118	0.7099	0.7274	0.7712
MTL on STJ (100%) + MTL (50%)$^{(2)}$	0.6610	0.7031	0.7128	0.7283	0.7706
MTL on STJ (100%) + MTL (80%)$^{(2)}$	0.7015	0.7136	0.7227	0.7459	**0.7977**
MTL on STJ (100%) + MTL (20%)$^{(2)}$	0.6357	0.6718	0.6658	0.7144	0.7342

Reflecting RMSE error range of 0–100 units and $R^2$.

Significant values are in [bold].

**Figure 4:**
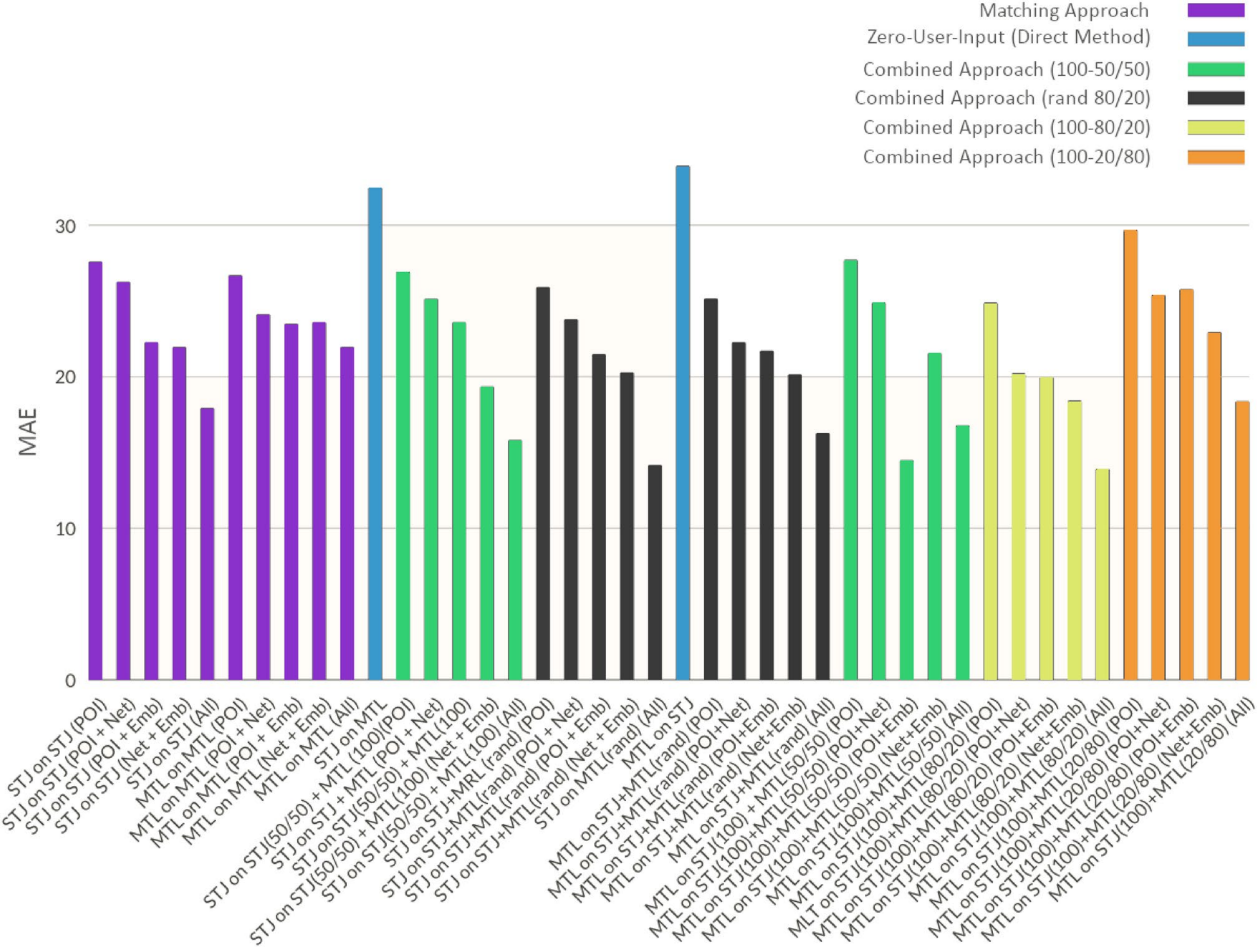
Exploration of 3 approaches (1) matching, (2) combined, (3) zero-user-input. Combined approach is extensively tested with various conditions. One such condition is the different ways of data split to better understand how the data affects the transfer of knowledge in transfer learning while being able to provide solid training and testing sets. Best performance was observed to be generated through a complete random selection into an 80–20 split. MTL on STJ reflects on the prediction of scores for Montréal based only on a model trained on St. John’s. MTL on STJ+MTL on the other hand reflects on the prediction of scores for Montréal based on a transfer-learned model on both St. John’s and Montréal. (Bar plot generated through matplotlib^45^ Version 3.4.3 from matplotlib.org).

As we explored in the background section, the goal of transfer learning is to take advantage of previously trained models, for instance models trained on the city of St. John’s NL in our previous works, to essentially extract knowledge that could be useful when applied to training new models (Combined approach) for new cities. However, transfer learning is also very useful to directly generate predictions for new cities without any more learning (Zero-user-input approach). Zero-user-input was our first transfer learning approach. Tables 4 and 5 and Fig. 4 highlight various experimentation performed on these 3 approaches over different feature combinations which show the Combined Approach with an 80–20 random split perform with the lowest MAE. We used our best model trained on data for the city of St. John’s using random forest to predict ALF-Score walkability for the city of Montréal (Fig. 2 top right). This resulted in a correlation of 0.4 compared to the predictions generated by a model that was trained purely on Montréal’s user data (Fig. 2 top left). Furthermore, our second approach of using previously trained models (MLP) towards training new MLP models (Fig. 2 bottom left) led a much higher correlation of 0.79 compared to the model only using the data from one city. We believe this promising model well utilizes the transferred knowledge in conjunction with the new learning gained by training over new data to identify additional patterns that may have not been fully captured by a model trained on a small set of user data from a single city.

**Figure 5:**
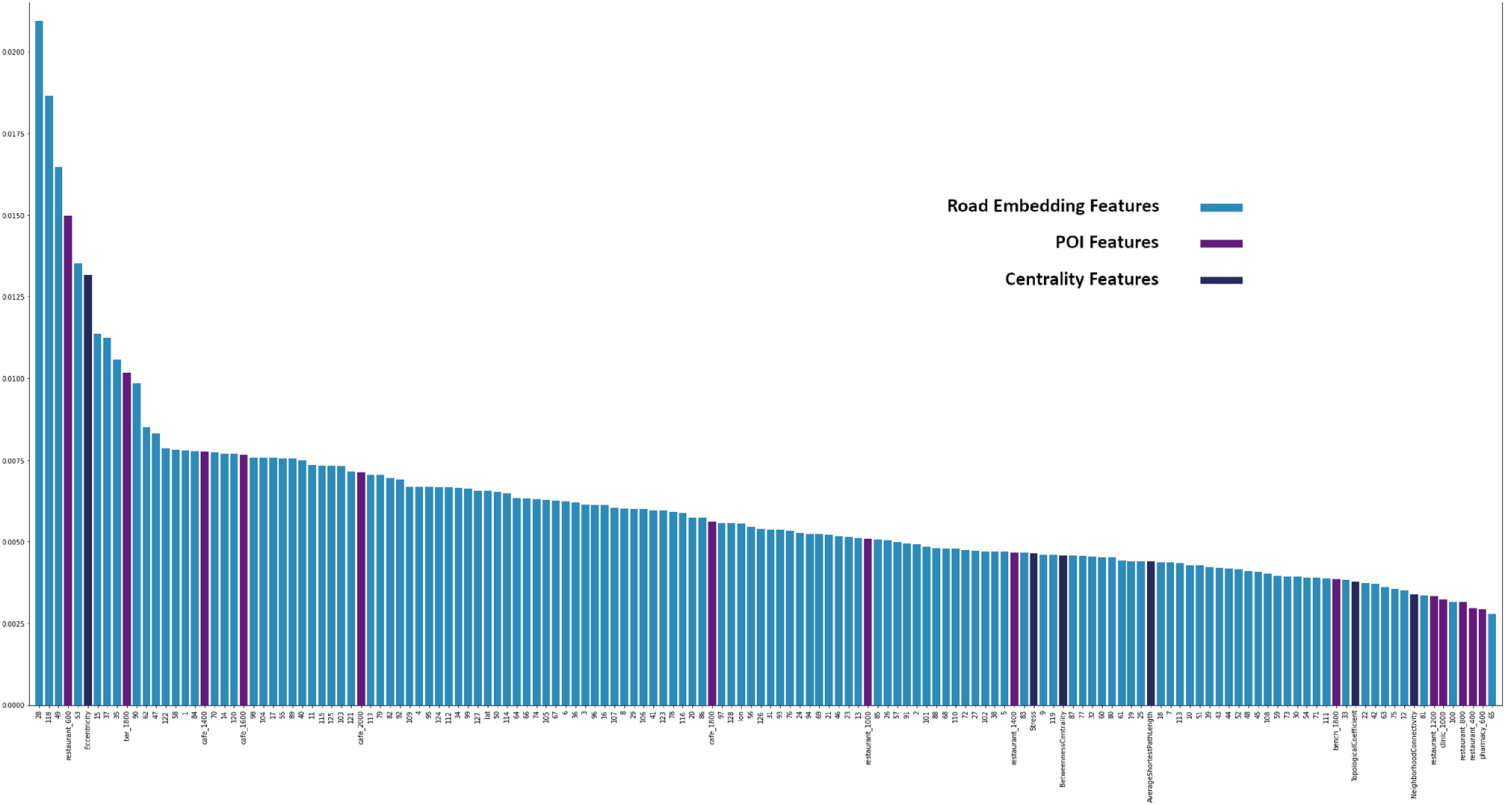
Top 150 features. While a noticeable difference is observed among the top 13 features, we can observe a steady trend among most embedding features. Embedding feature importance account for most of the feature importance. We can also observe that despite having the highest number of features (530) only a small number of POI features appear in the top 150 features. (Bar plot generated through matplotlib^45^ Version 3.4.3 from matplotlib.org).

In Fig. 5, we can observe that among the top 150 features (out of 668 features) 128 of them belong to the road embedding feature list (light blue) and account for all road embedding features. This is highlighting the importance of road embedding with regards to predicting walkability score based on user submitted ground-truth. Additionally, among top 150 features, only 14 belong to POIs (violet) which contribute to 530 features. Furthermore, among the top 150 features, 6 belong to centrality features (dark blue) out of the total 10 centrality features.

**Figure 6:**
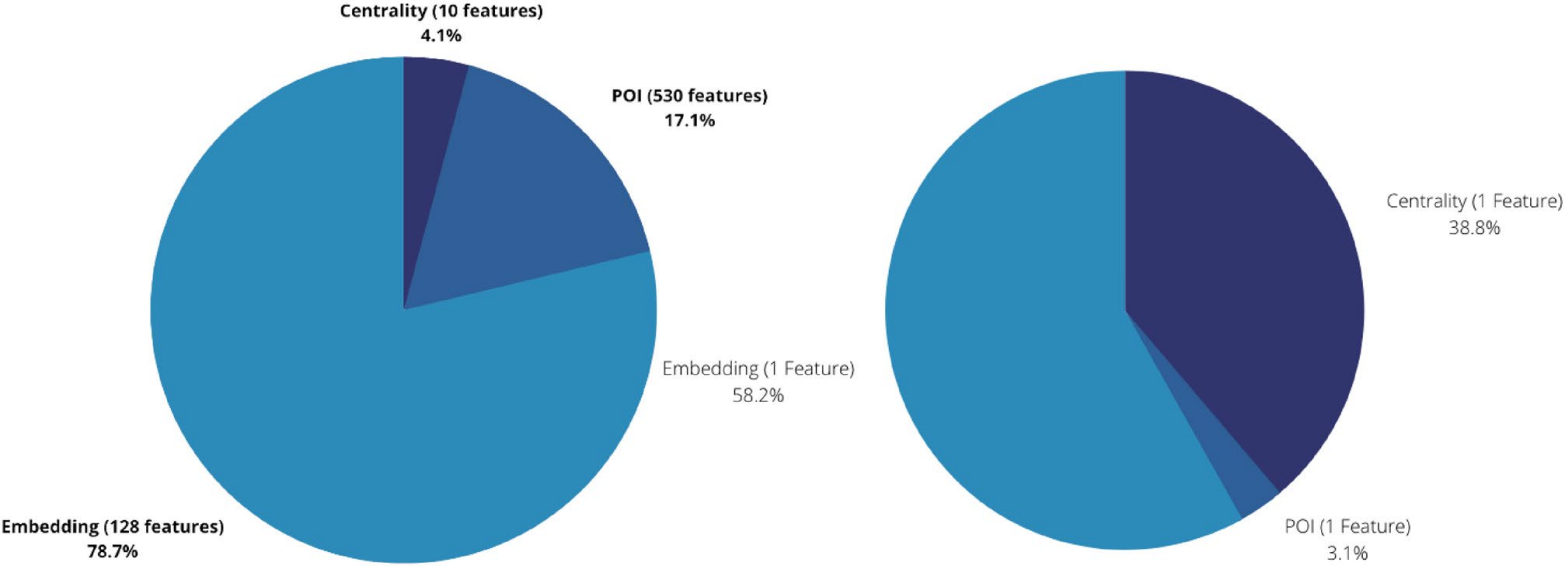
Total contribution to feature importance among 668 features is divided into three categories: (1) centrality, (2) POI, (3) road embedding. Left: road embedding, while contributing to only 19% of the total features, accounts for 78.7% of the total feature importance, while centrality features contribute to 4.1% and POI features to 17.1% of the total feature importance. Right: when normalized to individual feature importance, we can observe, the highest contribution is by embedding features where each feature contributes to 58.2% of the total embedding contribution of 78.7% where each centrality feature contributes to 38.8% of the total centrality feature importance of 4.1 while each POI feature contributes to only 3.1% of the total contributing feature importance of 17.1%. (Pie chart generated through matplotlib^45^ Version 3.4.3 from matplotlib.org).

The road embedding features account for 0.778486799 importance over 128 features while representing only 19% of the overall features. Figure 6 shows a general overview of feature importance distribution overall (left) and normalized for the number of features (right). We can observe the majority of the contribution to importance is by the road network features. The centrality features (Table 6) account for 0.039919843 importance over 10 features, and the POI features (Table 7) account for 0.169245465 importance over 530 features while representing over 79% of the features.

Eccentricity accounts for the highest centrality importance among the 10 features; however, it is contributing almost 33% to the overall centrality importance which is rather an important amount when considering there are 9 other centrality features as well. The highest ranked POI is 'restaurants' within 600 meters which contributes to almost 9% of all POI importance among 529 other POI features. Furthermore, it is very interesting to see 8 out of the top 10 POIs are either restaurants or cafes, while bars within 1800 meters and benches within 1800 meters amount to the remaining top 2 POIs. This points to the possibility of many people seeking to find places to socialize, with light entertainment and possibility to gather with friends and family. Especially, since the user data in this research was collected post COVID-19 pandemic, this may show an underlying effect of the pandemic’s isolation as to changing people’s priority and perception on places and the important value of socializing.

**Figure 7:**
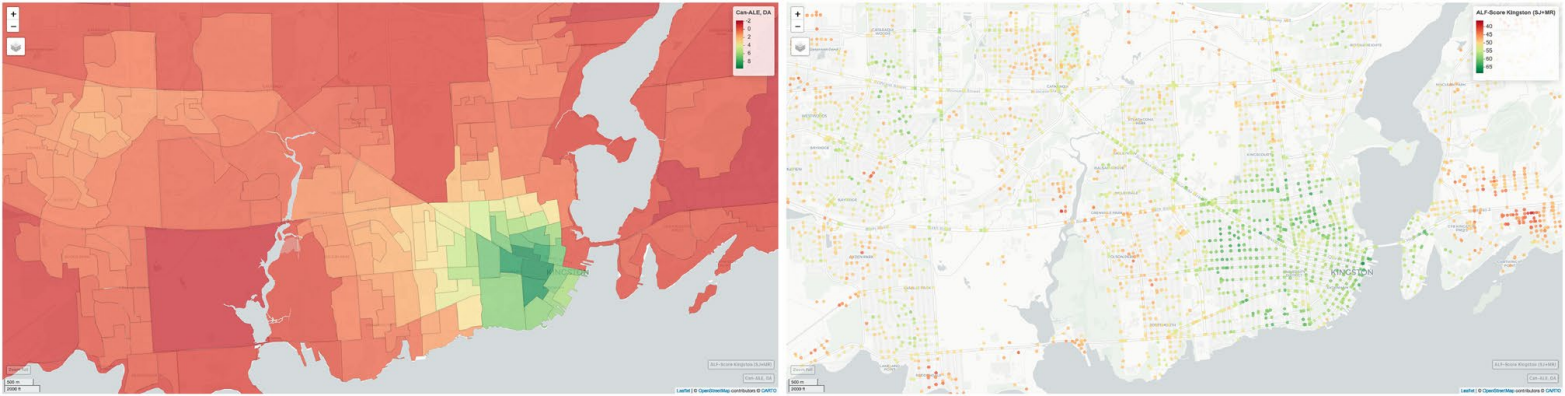
Left: Can-ALE for the city of Kingston, ON. Right: walkability results produced by ALF-Score++ for the city of Kingston, ON using the zero-user-input approach of a model trained through transfer learning based on user data from two cities of St. John’s and Montréal. The road network for Kingston maintains over 3400 nodes. ALF-Score’s walkability scores range between 0 and 100 units. This range can be adjusted if needed. (Maps generated through RStudio^44^ Version 1.2 using mapview package from rstudio.com).

The next step is to utilize the zero-user-input approach of the transfer learned model trained on the user data collected from the two cities of St. John’s NL and Montréal QC which have different structures, and applying this model directly to a third and a fourth cities of Kingston ON and Vancouver BC, which the model has never seen before, and generate ALF-Score walkability. In Fig. 7, we can see the ALF-Score walkability (right) compared to Can-ALE scores (left) for the city of Kingston, ON. At the first glance we can easily observe the variation in spatial resolution between these two methods with ALF-Score capturing the walkability of the region in a much greater depth. While Can-ALE shows some variation among different dissemination areas (DA), only the city center is highlighted with visible green and marked as walkable. Although ALF-Score++ agrees with Can-ALE with assigning higher walkability scores to the city center, the first major differentiator among the two is that in Can-ALE, higher walkability is given to the central and highly populated areas of the city center whereas in ALF-Score++, while central region is ranked with higher walkability, ALF-Score++ recognizes the core as slightly less walkable compared to locations surrounding the core of the city center. Specifically, ALF-Score++ favours waterfront walkways and paths as more walkable as opposed to Can-ALE. For instance, the area near to Leon’s Centre on Ontario Street is known to be a walkable area and is ranked with high walkability by ALF-Score’s zero-user-input approach, whereas it is ranked with a significantly lower walkability score by Can-ALE.

**Table 6:** Feature importance for all centrality features (10 features in total) which contribute to 4.1% of the total feature importance.

Feature	Importance
Eccentricity	0.01316184
Stress	0.004649347
Betweenness centrality	0.004590322
Average shortest path length	0.0043923
Topological coefficient	0.003773664
Neighborhood connectivity	0.003381009
Radiality	0.002233024
Closeness centrality	0.001581954
Clustering coefficient	0.001386535
Degree	0.000191574

**Table 7:** Feature importance for top 10 (from 530) POI features.

Feature	Importance
restaurant_600	0.014984423
bar_1800	0.010154083
cafe_1400	0.007755144
cafe_1600	0.007659399
cafe_2000	0.007125045
cafe_1800	0.005620239
restaurant_1000	0.005089702
restaurant_1400	0.004664054
restaurant_1400	0.004664054
bench_1800	0.003845234

The entire 530 features contribute to 17.1% of the total feature importance.

Additionally, ALF-Score captured a cluster of greener/more walkable spots close to student's housing and living quarters near Queen’s University. While this area is popular among many students, faculty and other members of the public, Can-ALE was unable to capture it due to its area-based structure and lower spatial resolution. Moreover, we observed various other areas that ALF-Score++ ranked as walkable whereas Can-ALE failed to capture their actual walkability due to it’s lower resolution and granularity. For instance, the Division St.—Dalton Ave.—Benson St. region (which falls under multiple DAs) is ranked with low walkability scores by Can-ALE whereas ALF-Score captured and distributed much more refined and relatable walkability scores to varying spots where there are many restaurants, stores and other popular places. Furthermore, the walkability of Point Frederick Peninsula (across the LaSalle Causeway bridge) is in the red zone of Can-ALE's scores while ALF-Score suggests the opposite for the region. This region houses multiple military campuses with varying facilities and is deemed walkable.

**Figure 8:**
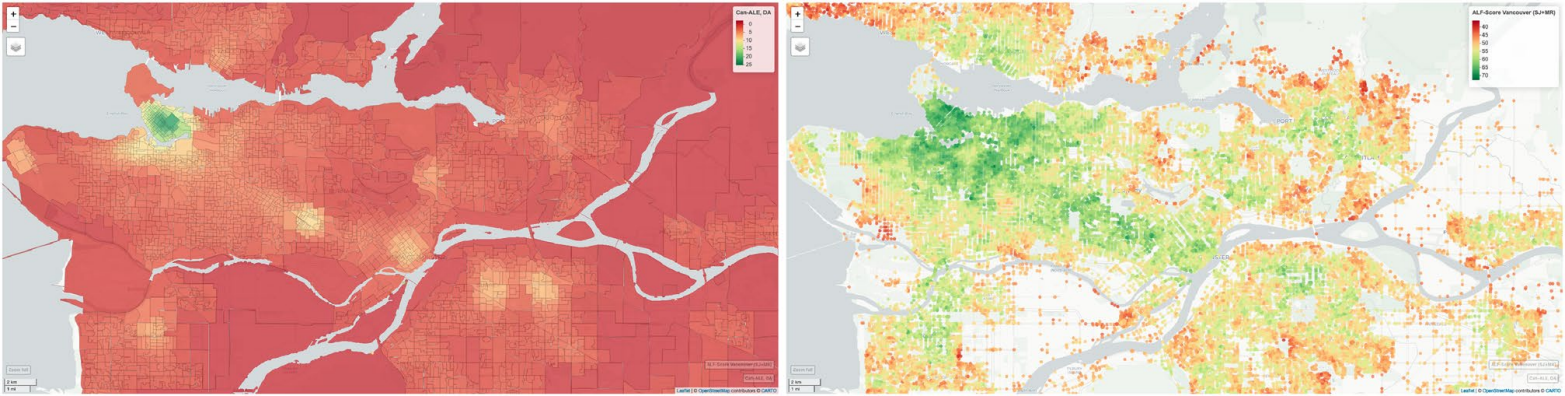
Left: Can-ALE for the city of Vancouver, BC. Right: walkability results produced by ALF-Score++ for the city of Vancouver, BC using the zero-user-input approach of a model trained through transfer learning based on user data from two cities of St. John’s and Montréal. The road network for Vancouver maintains over 45 thousand nodes. (Maps generated through RStudio^44^ Version 1.2 using mapview package from rstudio.com).

Figure 8 shows the ALF-Score++ walkability (right) compared to Can-ALE scores (left) for the city of Vancouver, BC. The ALF-Score++ for this region is generated based on a zero-user-input approach and similar to ALF-Score++ for Kingston, we can observe high spatial resolution as opposed to Can-ALE’s low spatial resolution for the same area. To look further into this region, we can start by observing the University of British Columbia campus where Can-ALE highlights the inner campus area (left side) with light orange while the outer campus area (right side) remains darker orange. ALF-Score++ picks up on the fact that the right area should be more walkable due to bus stops and various facilities commonly used by students and staff. Additionally, North Vancouver’s walkability appears not to have been captured by Can-ALE where its walkability for the region is ranging between dark orange and red. In contrast, ALF-Score better captures various popular areas in North Vancouver that are walkable. Furthermore, the walkability for the Richmond area is barely captured by Can-ALE with mostly dark orange and red walkability. ALF-Score++ on the other hand is able to capture various walkable areas in that region. An interesting observation here is the similarity with zero-user-input walkability data generated for the city of Kingston. Can-ALE typically marks areas close to water as less walkable whereas ALF-Score++ tends to object. ALF-Score++’s results are positively associated to our collective knowledge of Vancouver and Kingston. We can observe that ALF-Score++ is utilizing its transferability capabilities to better understand the city structures and find patterns in various associated data to generate zero-user-input walkability scores for virtually any location on the road.

As observed earlier, the combination of user data from just two cities of St. John’s and Montréal allowed us to generate accurate walkability scores for cities never seen by our algorithms. It is our observation that transfer learning works well in this application even with a small set of user data. Additionally, we believe as we accumulate more user data, our algorithms will be able to better capture various patterns in the data leading to an improved accuracy.

In this research, we were also able to show ALF-Score++’s pipeline is scalable as data size increases. The pipeline was optimized to perform well while processing, training and predicting walkability scores for small and large cities alike. One of the major enhancements to the pipeline was improving the GLEPO algorithm such that the processing time is reduced. This reduction process went through multiple stages. In our initial trials every iteration of GLEPO took approximately 17 min on a personal MacBook configured with a 2.2 GHz dual-core Intel Core i7 (Turbo Boost up to 3.2 GHz) with 4 MB shared L3 cache and 8 GB of 1600 MHz LPDDR3 on-board memory. Over a typical run of the algorithm, we went through approximately 50 iterations totaling to over 14 h of operation. We found this to be unreasonable. In the final stage of this improvement we were able to process the same data over the same computer through the newly updated ALF-Score++ pipeline in just under 3 min per iteration, a reduction of almost 6 fold. A GLEPO run of 50 iterations will now only take 2.5 h. Additionally, after rigorous experimentations and tests, we determined the optimal number of iterations desired for GLEPO algorithm is 50 iterations while the minimum required number of iterations to achieve convergence is 30 iterations leading to a successful completion of the process within 1.5 h.

## Discussion

**Figure 9:**
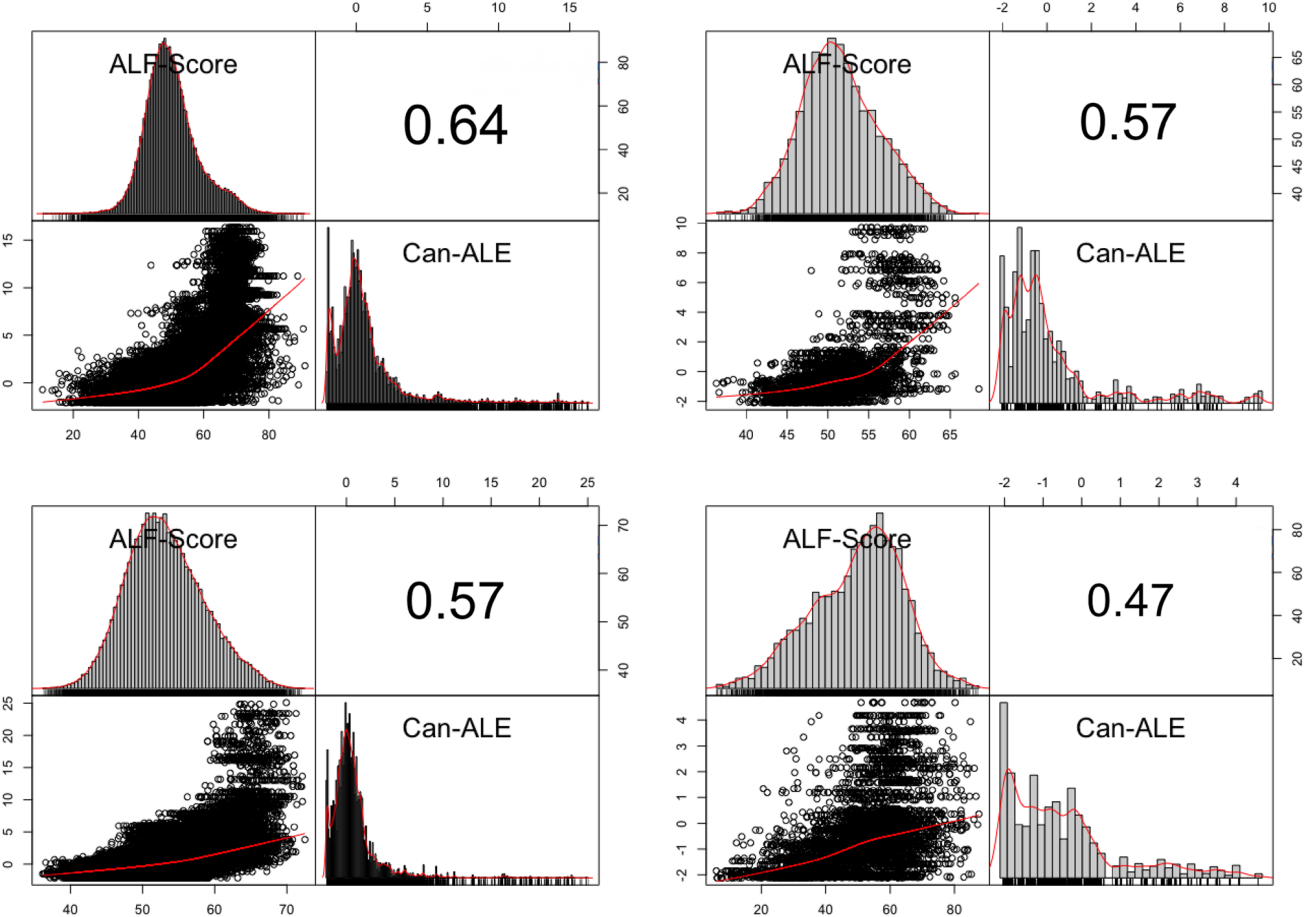
Correlation between ALF-Score++ and Can-ALE for four different cities. Top left: Montréal QC, Top right: Kingston ON, Bottom left: Vancouver BC, Bottom right: St. John’s NL. (Correlation figures generated through RStudio^44^ Version 1.2 using PerformanceAnalytics package from rstudio.com).

The goal of the overall research is aimed to explore how machine learning can be applied to the spatial domain with application in public health through generating relevant and meaningful walkability scores with high spatial resolution based on a very small set of users' opinions. In this paper, we showed that ALF-Score++’s pipeline is fully capable of scaling up and down to match the data based on the size of the city and user opinion data and still perform in a reasonably timely manner. Additionally, since the computational complexity of the pipeline is $O(n^2)$, we expect processing larger cities will perform reasonably and within the expected parameters. We were able to show that ALF-Score++ can process and generate models for the city of Montréal QC which is almost 16 times larger than St. John’s NL within a timely fashion without requiring any extended resources while these models are capable of producing walkability scores with high spatial resolution compared to that of Can-ALE. Figure 9 shows a comparison between ALF-Score++ walkability scores and Can-ALE walkability scores for four different cities in Canada.

Moreover, we saw the power of transferability giving us the upper hand to transfer the knowledge learned from small cities to predict accurate walkability scores for much larger cities. This leads to many advantages such as reduced resource requirement and reduced processing time while increasing the flexibility and applicability of our trained models. Furthermore, its application of zero-user-input transfer learning proved to be a huge success in predicting walkability scores for cities never seen by the algorithm before and without any prior knowledge about them while utilizing previously learned information and patterns. Of note, the transfer learning was able to capture both the relative and absolute differences between cities in terms of walkability. For example, the range of walkability scores assigned to Kingston based on the transfer learning was 30–70, whereas Vancouver scores based on transfer learning ranged from 30 to 80, when St. John’s and Montréal were used as training cities. Developing measures that capture both relative and absolute differences in cities has been an on going challenge, that transfer learning may be able to solve.

We also observed how adding a small set of user opinion from a different region can lead to a much higher pattern recognition by the models while allowing a better generalization of these models. This generalization can therefore help capture various common patterns found in different cities without any actual prior knowledge about them.

Similar to many machine learning tasks, we believe ALF-Score++’s pipeline can benefit from and train more accurate models with more data. For instance, collecting small user data samples across various cities and towns could cover a much more diverse set of user demographics, users' opinions, patterns, city and road structures, leading to a well-generalized model applicable to virtually any loca tion on the map . Given enough user information from a few select key cities in Canada (cities with varying structure and sizes), the models generated through transfer learning of this data will be able to provide accurate scores anywhere in Canada without the need to train models on data for every individual city leading to a global model. To generate new models to add new data, one does not need to rerun the entire process on the entire data sets. All that is required is to transfer the knowledge from previously trained models (which can be transfer learned models themselves) and only run a smaller transfer learning task on the newly collected data. We also believe ALF-Score++ pipeline can be adjusted to be fully capable of continuous learning. This could be particularly important as changes to road networks are detected. Our network-based approach combined with continuous transfer learning can help our models detect patterns associated with various regions, types of road and user demographics and provide accurate predictions for new roads and structures never seen by the model.

As we went through the predictive process, a variation was observed between the performance of shallow and deep models. Throughout ALF-Score, random forest (a shallow model) was the preferred technique since (1) it performed best across all other techniques (shallow and deep) achieving an MAE error as low as 4.56 units, (2) its simplicity and powerful approach, (3) faster processing and predictions compared to MLP. Although, MLP (a deep model) is the main technique used in ALF-Score++ since deep models are preferred when it comes to transfer learning due to their layered structure. The lowest error was achieved using MLP at 13.87 units.

A side-effect of transfer learning is its generalization. Each city will have its own range of walkability. Small cities may have a smaller range of walkability whereas larger cities may have a wider range of walkability. When models trained on small and large cities are combined through transfer learning, the newly trained model will be more generalized. Although this generalization is very important to be able to take a zero-user-input approach to generate walkability scores for cities never seen by the algorithms, one must keep in mind that a balance of data must be maintained. As observed earlier (in Montréal’s results), applying a model trained only on a small city might not capture the varying patterns of a larger city and vise versa. It is important to ensure the transfer learning process maintains a good balance of user data for training, such as using user data for a small and a large city to build the base model with transferability capabilities. These small samples can prove to be invaluable in improving the overall quality and accuracy of pattern detection and prediction. Moreover, to further address the generalization phenomenon happening during the transfer learning phase, we can utilize ALF-Score’s personalization extension (ALF-Score+) to create personalized and transferable models that are generalized to various city structure patterns, yet personalized to specific individual profile clusters. As demonstrated in our previous work, ALF-Score+^19^, concentrating on specific profile clusters which contain users with similar opinion towards walkability ranking, will significantly improve the overall accuracy of each personalized model.

For a more direct comparison, ALF-Score++ is able to provide a comprehensive and well-distributed range of walkability scores to each node on the road across cities as opposed to area-based walkability measures such as Can-ALE^17^ that provide low spatial resolution scores associated with large DA areas. Moreover, the inclusion of predictability and crowd-sourced user opinion significantly changes the way walkability scores are measured by incorporating users’ perspectives as ground truth to more accurately reflect individuals’ points of view and build a significantly stronger model over time. Since ALF-Score is the first known predictive walkability measure and since walkability is subjective, it is difficult to directly compare its scores with that of others. But in a broader spectrum, ALF-Score and its extensions, ALF-Score+ and ALF-Score++, have introduced some new approaches such as predictability, personalization and transferability of walkability that no other walkability measure, as of the time of this research, offer.

We believe ALF-Score and its various extensions such as ALF-Score+ and ALF-Score++ can be very beneficial and act as very powerful tools for many people from various backgrounds working on different domains. Although ALF-Score can produce results specific to various parameters, such as demographics to provide personalized walkability scores, ALF-Score’s pipeline takes a generalized approach instead to allow for various issues that may not be related to walking or walkability be addressed using this method. For instance, bikeability^46^, school friendliness, transit friendliness^47^, or even POI specialties based on different demographics and perceptions. Moreover, the pipeline is capable of handling wide variety of features as well as other types of networks instead of road network. For example, subway networks. At its core, ALF-Score requires a vector of user-based ground truth labels alongside a list of features and uses a dedicated web-tool to crowd-source this user-based data. However, ALF-Score’s pipeline follows a black box system and works with any compatible input data regardless of how they were collected, prepared or processed. The ground truth data can be processed according to individual researchers’ needs and this step can be bypassed in the pipeline. Although walkability scores generated by ALF-Score and its extensions rely on user labels and road network data, the generalization offered by ALF-Score's pipeline can be further distilled to beyond road networks. Road network data is treated as any other features and can be replaced with an appropriate feature based on the issue at hand and the research requirements. We genuinely believe that ALF-Score opens the door to many possibilities well beyond the scope covered in this research.

## Data Availability

The datasets generated during and/or analysed during this study are available through the Harvard Dataverse repository located at https://doi.org/10.7910/DVN/WBQXXZ.
